# Induction of Antitumor Immunity by Exosomes Isolated from Cryopreserved Cord Blood Monocyte-Derived Dendritic Cells

**DOI:** 10.3390/ijms21051834

**Published:** 2020-03-06

**Authors:** Uyen Thi Trang Than, Huyen Thi Le, Diem Huong Hoang, Xuan-Hung Nguyen, Cuong Thi Pham, Khanh Thi Van Bui, Hue Thi Hong Bui, Phong Van Nguyen, Tu Dac Nguyen, Thu Thi Hoai Do, Thao Thi Chu, Anh Viet Bui, Liem Thanh Nguyen, Nhung Thi My Hoang

**Affiliations:** 1 Vinmec Research Institute of Stem Cell and Gene Technology (VRISG), Vinmec Healthcare system, Hanoi, 458 Minh Khai, Hanoi 10000, Vietnam; v.uyenttt@vinmec.com (U.T.T.T.); huyenlengoc94@gmail.com (H.T.L.); hoanghuongdiem95@gmail.com (D.H.H.); v.hungnx1@vinmec.com (X.-H.N.); phamcuong1997na@gmail.com (C.T.P.); v.huebth@vinmec.com (H.T.H.B.); v.liemnt@vinmec.com (L.T.N.); 2 College of Health Sciences, VinUniversity, Hanoi, Vinhomes Ocean Park, Hanoi 10000, Vietnam; 3 VNU University of Science, Vietnam National University, Hanoi, 334 Nguyen Trai, Hanoi 10000, Vietnam; buithivankhanh@hus.edu.vn (K.T.V.B.); dothihoaithu_t58@hus.edu.vn (T.T.H.D.); 4 Vinmec HiTech Center, Vinmec Healthcare System, Hanoi, 458 Minh Khai, Hanoi 10000, Vietnam; v.phongnv5@vinmec.com (P.V.N.); v.tund5@vinmec.com (T.D.N.); v.thaoct@vinmec.com (T.T.C.); v.anhbv@vinmec.com (A.V.B.)

**Keywords:** dendritic cells, exosome, dendritic cell vaccination, tumor cell lysate-specific-CD8^+^ T cells, CD3^+^Vγ9 T cells

## Abstract

(1) Background: Dendritic cell (DC) vaccination has shown outstanding achievements in cancer treatment, although it still has some adverse side effects. Vaccination with DC-derived exosomes has been thought to overcome the side effects of the parental DCs. (2) Method: We performed the experiments to check the ability of cryopreserved umbilical cord blood mononuclear cell-derived DCs (cryo CBMDCs) and their exosomes to prime allogeneic T cell proliferation and allogeneic peripheral blood mononuclear cell (alloPBMCs) cytotoxicity against A549 lung cancer cells. (3) Results: We found that both lung tumor cell lysate-pulsed DCs and their exosomes could induce allogeneic T cell proliferation. Moreover, alloPBMCs primed with tumor cell lysate-pulsed DCs and their exosomes have a greater cytotoxic activity against A549 cells compared to unprimed cells and cells primed with unpulsed DCs and their exosomes. (4) Conclusion: Tumor cell lysate-pulsed DCs and their exosomes should be considered to develop into a novel immunotherapeutic strategy—e.g., vaccines—for patients with lung cancer. Our results also suggested that cryo umbilical cord blood mononuclear cells source, which is a readily and available source, is effective for generation of allogeneic DCs and their exosomes will be material for vaccinating against cancer.

## 1. Introduction

Dendritic cells (DCs) are professional antigen-presenting cells with a powerful capability of activating tumor-specific T cell responses [1,2]. Antigen-loaded DCs have been developed as vaccines to break the immune tolerance within the tumor microenvironment and effectively promote T cell-mediated anti-tumor immune responses. However, the limited response rate, undefined composition, and chemokine gradient dependency of autologous DC (autoDC)-based vaccines limit their broad applications [1,2]. Therefore, allogeneic DCs (alloDCs) and DC-derived exosomes have been developed as a novel cancer vaccine strategy to overcome the limitations of autoDC-based strategies [1,2,3,4].

Compared to autoDCs, alloDCs are more capable of inducing specific immune responses because they can more broadly activate CD8^+^ T cell immunity, including tumor-responsive T cells and broad inflammatory responses by polyclonal stimulation of allogeneic T cells [5,6]. In addition, alloDCs can be produced at a large scale for patients, thereby ensuring vaccine stability and standardization of the DC vaccine quality. Various clinical trials, conducted to evaluate the safety and efficacy of alloDC-based tumor vaccination [7,8,9,10,11,12,13,14,15], have demonstrated the ability of the vaccine to activate tumor-specific T cells. In particular, a phase I clinical study from Loosdrecht et al. demonstrated that administering an alloDC cell line (named the DCP-001 cell line) to elderly patients with acute myeloid leukemia was safe and effective, and generated both cellular and humoral immune responses [15].

Exosomes are nanometric vesicles with an average diameter of 30 to 150 nm that are packed with diverse biomolecules, such as lipids, proteins, and small RNAs (including microRNAs, messenger RNAs, transfer RNAs, and ribosomal RNAs) [16,17,18]. Mouse bone marrow-derived DCs, human primary monocyte-derived DCs, and DC cell lines have all been reported to release exosomes into the extracellular environment [19,20]. Exosomes possess not only the typical characteristics of DCs in presenting antigens to T cells via surface expression of functional major histocompatibility complex–peptide complexes but they can also present other costimulatory molecules and components [21,22]. Therefore, DC-derived exosomes can induce tumor rejection and have distinct advantages over cell-based immunotherapies, including DCs, and has been used in many clinical trials [23,24,25].

AlloDCs and autoDC vaccination and their exosomes have been applied in several clinical trials, but to date there is no reports using cord blood monocyte-derived DCs and their exosomes in cancer treatment [7,8,9,10,11,12,13,14,15,23,24,25]. Banks of cryopreserved umbilical cord blood mononuclear (cryo CBMCs) has been widely applied in all over the world and could be a reliable, readily available, and efficient source of DCs for anticancer vaccination [20,21,26].

In this study, we aimed to evaluate the immunogenicity of alloDCs differentiated from cryo CBMCs and their isolated exosomes. Both A549 lung tumor cell lysate-pulsed DCs and their exosomes induced allogeneic T cell proliferation and allogeneic peripheral blood mononuclear cell (alloPBMCs)-mediated cytotoxicity against A549 lung cancer cells. We demonstrated that DCs pulsed with A549-cell lysates represented the best potential candidate for inducing T cell proliferation and alloPBMCs anti-tumor cytotoxicity. We also found that exosomes derived from A549 lysate-pulsed DCs and un-pulsed DCs elicited moderate T cell activation and anti-cancer effects.

## 2. Results

### 2.1. Successful Generation of Cryopreserved Umbilical Cord Blood Mononuclear Cell-Derived DCs (Cryo CBMDCs)

**Figure 1:**
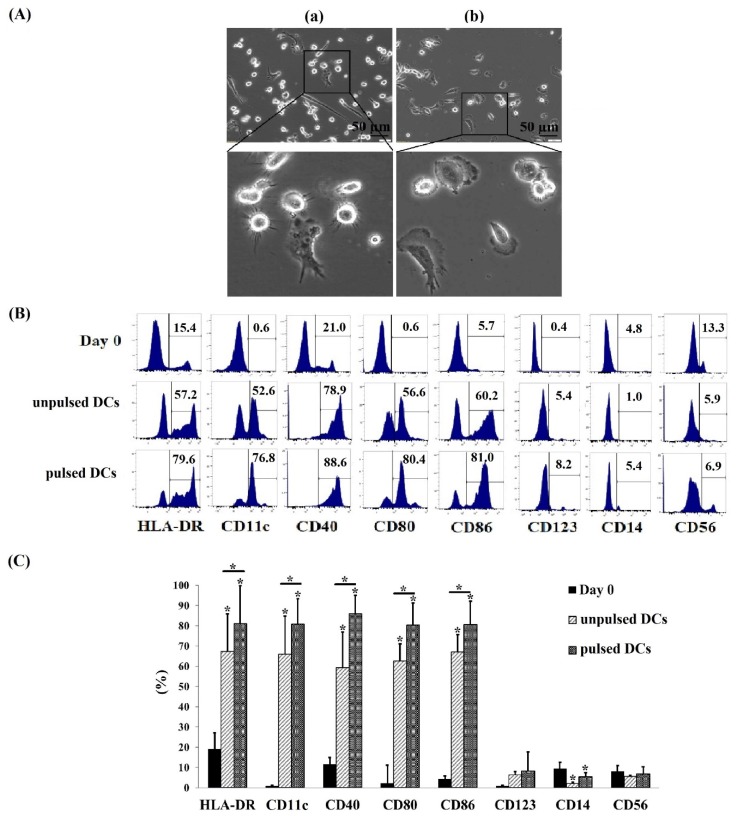
Dendritic cells (DCs) differentiated from human cord blood (CB) monocyte cells. (**A**) Morphology of DCs differentiated from CB monocyte cells in the presence of granulocyte-macrophage colony-stimulating factor (GM-CSF), IL-4, and tumor necrosis factor (TNF)-α. (**Aa**) Morphology of DCs which were pulsed with A549 protein; (**Ab**) morphology of DCs which were not pulsed with A549 protein. The DCs exhibited many dendrites protruding from the cell bodies, which are characteristic of mature, pulsed DCs. (**B**) The percentages (%) of DC-surface markers expressed in pulsed DCs and unpulsed DCs. The pulsed DCs expressed high levels of dendritic surface markers including HLA-DR, CD11c, CD40, CD80, and CD86 while lower levels of these markers were detected on unpulsed DC. (**C**) The comparison of DC surface marker expression between unpulsed DCs and pulsed DCs. Noted that more than 80% of cells expressed DC markers in pulsed DCs population, meanwhile this value was more than 59.0% in unpulsed DCs. Data was presented as mean ± SD in quadruplicate cultures (* *p* < 0.05). pulsed DCs: A549 tumor cell lysate-pulsed DCs; unpulsed DCs: A549 tumor cell lysate-unpulsed DCs.

DCs were generated from cryo CBMCs by inducing differentiation in medium containing granulocyte-macrophage colony-stimulating factor (GM-CSF) and interleukin-4 (IL-4). After three days of cell culture, adherent and non-adherent DCs were observed that showed a heterogeneity of the cell size. By day seven, all cells were observed and DCs pulsed with A549-derived protein exhibited typical features of mature cells with mainly long and thin dendrites, fewer short dendrites, and a star-shaped morphology (Figure 1(Aa)). On the other hand, DCs not pulsed with A549-derived protein exhibited shorter dendrites compared to the A549-derived protein-pulsed DCs (Figure 1(Ab)).

To analyze the surface phenotype of pulsed and ulpulsed DCs, the cells were characterized by flow cytometry using fluorescently labeled antibodies against human leukocyte antigen D-related (HLA-DR), cluster of differentiation (CD)14, CD11c, CD40, CD56, CD80, CD86, and CD123. Compared to CBMCs at day 0 of cell culture, pulsed DCs and unpulsed DCs at day seven consistently showed an increase expression level of DC markers such as HLA-DR, CD11c, CD40, CD80, and CD86, and low expression of monocyte markers such as CD123, CD144, and CD56 (*p* < 0.05) (Figure 1B). The percentage of cells expressing DC markers was significant higher in pulsed DCs when compared to unpulsed ones (*p* < 0.05). The pulsed DC population expressed DC markers were high on most cell culture such as HLA-DR: 81.0 ± 18.7%, CD11c: 80.9 ± 12.4%, CD80: 80.4 ± 29.1%, CD40: 85.9 ± 9.1%, and CD86: 80.6 ± 16.8% (Figure 1C). This indicated that mature DCs have been generated successfully from cryo CBMCs.

### 2.2. Typical Characteristics of DC-Derived Exosomes

**Figure 2:**
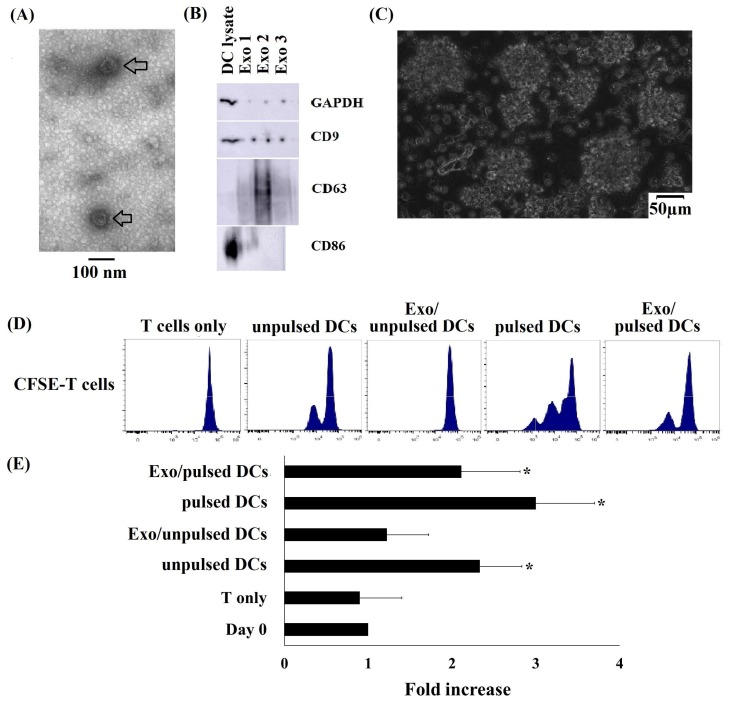
DCs and their exosomes induced allogeneic T cell proliferation. (**A**) A cup-shaped morphology was observed for pulsed DC-derived exosomes by TEM. (**B**) Pulsed DC-derived exosomes expressed CD9, CD63, and CD86. (**C**) AlloT cells grew as clumps when incubated with pulsed DCs. (**D**) Carboxyfluorescein succinimidyl ester (CSFE)-stained T cells proliferated during a seven-day incubation with pulsed DCs, unpulsed DCs and exosomes isolated from DCs. Untreated T cells or T cells incubated with exosomes isolated from Exo/unpulsed DCs did not divide. (**E**) The number of T cells increased highest in the treatment with pulsed DCs, followed by Exo/pulsed DCs, and unpulsed DCs. Exo/unpulsed DCs showed no significant effect on alloT cell proliferation. Data was presented as mean ± SD in quadruplicate cultures (* *p* < 0.05). Exo1: pulsed DC-derived exosome sample 1; Exo2: pulsed DC-derived exosome sample 2; Exo3: pulsed DC-derived exosome sample 3. DCs: dendritic cells; pulsed DCs: A549 tumor cell lysate-pulsed DCs; unpulsed DCs: A549 tumor cell lysate-unpulsed DCs; Exo/unpulsed DCs: exosomes isolated from unpulsed DCs; Exo/pulsed DCs: exosomes isolated from pulsed DCs.

To analyze the morphology characteristics of exosomes released by pulsed DCs and unpulsed DCs, exosomes from these cells subjected to negative staining and visualized using transmission electron microscopy (TEM). The images showed that the DC-derived exosomes had a cup-shaped morphology and a nanometer-scale size (Figure 2A, arrow). Additionally, protein immunoblot has been applied to detect exosomal marker expression. Data indicated that exosomes released from DCs expressed CD9 and CD63 which are considered as exosome markers (Figure 2B). Besides, CD86 was a DC marker was detected in both DCs and DC-derived exosomes. However, the expression of CD86 was very different that very abundant in DCs but very little in DC-derived exosomes (Figure 2B). This data indicates that DC-derived exosomes carry the DC characteristic.

### 2.3. Cryo CBMDCs and their Exosomes Induced the Proliferation of Allogeneic T Cells

T lymphocytes were isolated from healthy donors’ peripheral blood using a CD3 Microbeads Positive Selection Kit. The isolated T cells were highly pure, as indicated by the calculation that 98.7 ± 1.2% of the cells expressed CD3^+^. To determine the capacity of DCs and DC-derived exosomes to induce allogeneic T cell proliferation, CFSE-stained T cells were incubated with DCs or DC-derived exosomes. Results showed that T cells incubated with pulsed DCs grew as clumps with a morphology characteristic of T cells growing in logarithmic phase (Figure 2C). The proliferative responses of T cells were determined by measuring the CFSE fluorescence intensity. The data showed that pulsed DC-induced T cells had a highest proliferation rate with a three-fold increase in the cell number during seven days of culture when compared to that of non-induced control T cells. There was a lower rate of T cell proliferation observed in the T cell group treated with unpulsed DCs, only two times higher than the control T cell. Interestingly, pulsed DC-derived exosomes (Exo/pulsed DCs) increased the number of T cells by 2.3 folds, while there was no division to increase cell number observed in the T cell group with the stimulation of ulpulsed DCs-derived exosomes (Exo/ulpulsed DCs) (Figure 2D,E).

**Figure 3:**
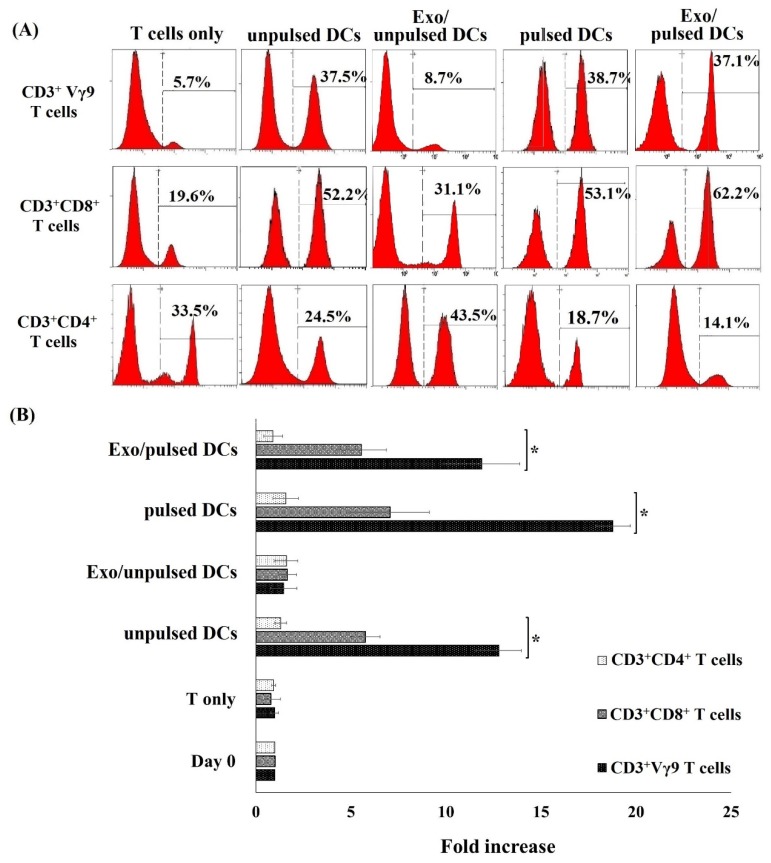
DCs and their exosomes induced the proliferation of allogeneic T cell subpopulation. (**A**) After incubation with pulsed DCs, unpulsed DCs, and Exo/pulsed DCs, the percentage of both CD3^+^Vγ9 T cells and CD3^+^CD8^+^ T cells increased compared to T cells without such treatments. (**B**) Fold increase in the number of CD3^+^Vγ9 T cell and CD3^+^CD8^+^ T cell induced by DCs and their exosomes. Both pulsed DCs and their exosomes successfully induced CD3^+^Vγ9 T cell and CD3^+^CD8^+^ T cell proliferation, meanwhile the number of CD3^+^CD4^+^ T cells did not increase. Exo/pulsed DCs could induce the percentage of these cell sub populations similar to unpulsed DCs did, but exosomes isolated from unpulsed DCs did not. Data was presented as mean ± SD in quadruplicate cultures (* *p* < 0.05). DCs: dendritic cells; pulsed DCs: A549 tumor cell lysate-pulsed DCs; unpulsed DCs: A549 tumor cell lysate-unpulsed DCs; Exo/unpulsed DCs: exosomes isolated from unpulsed DCs; Exo/pulsed DCs: exosomes isolated from pulsed DCs.

Interestingly, among the proliferating T cell populations, CD3^+^Vγ9 T cell subpopulation was the most effective in responding to stimulations, for examples increased from 5.2 ± 1.7% (day 0) to 31.2 ± 3.1%, 35.7 ± 7.4%, and 32.1 ± 8.3% (day seven) in responding to unpulsed DCs, pulsed DC, and Exo/pulsed DCs, respectively (Figure 3A, Table 1). Additionally, the proportion of CD3^+^CD8^+^ T cells increased from 25.8 ± 4.2% (day 0) to 55.3 ± 2.4%, 52.7 ± 5.1%, and 58.9 ± 4.6%, respectively in responding to unpulsed DCs, pulsed DC, and Exo/pulsed DCs (Figure 3A, Table 1). Thus, the average proliferation of CD3^+^Vγ9 T cells and CD3^+^CD8^+^ T cells under all treatment conditions increased by more than 11.8 ± 1.5 and 6.0 ± 1.6-fold, respectively, compared to the control cells (Figure 3B). There was no significant difference in T cell proliferation induction capacity among pulsed DCs, unpulsed DCs, and Exo/pulsed DC (Figure 3B, *p* ˃ 0.05). On the contrary to CD3^+^Vγ9 T cell and CD3^+^CD8^+^ T cell populations, CD3^+^CD4^+^ T cells did not proliferate in responding to stimulatory factors of any pulsed DC, unpulsed DC or exosomes from DCs. Besides, Exo/unpulsed DCs showed no effect on the proliferation of T cell or any T cell subset (Table 1, Figure 3). These data indicated that pulsed DCs, unpulsed DCs and pulsed DC-derived exosomes all had important influence on T cell proliferation but the response of T cells to the stimulatory factors depends on T cell subpopulation.

### 2.4. Greater Cytotoxic Activity of Allogeneic PBMCs and Allogeneic T Cells Primed with DCs and their Exosomes on A549 Cancer Cells

**Figure 4:**
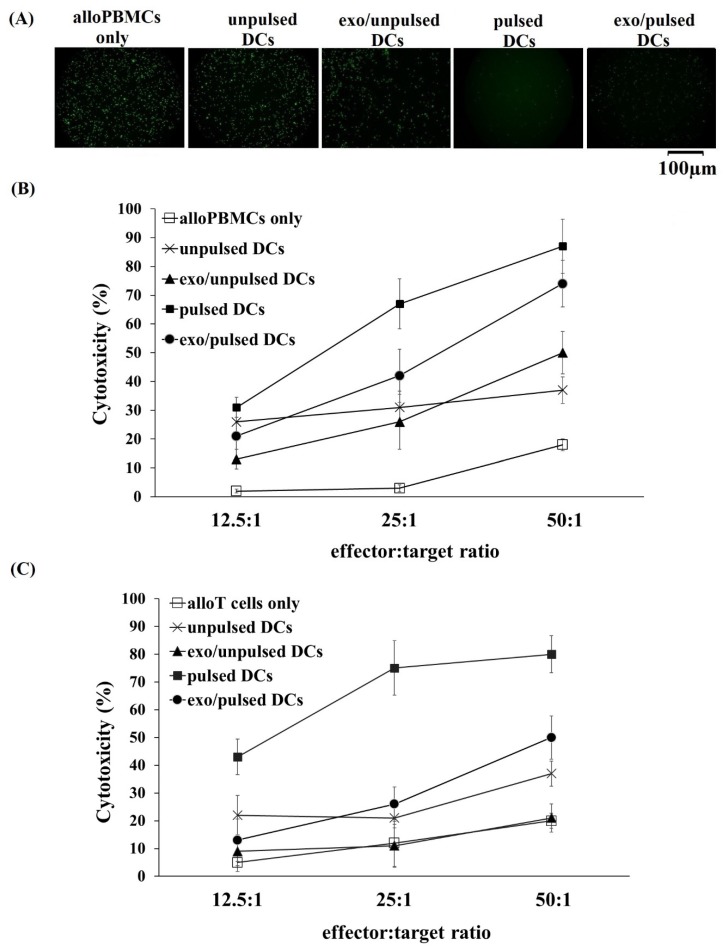
Cytotoxic activity of alloPBMCs in A549 lung cancer cells induced by DCs and DC-derived exosomes. (**A**) Calcein-AM-labeled A549 cancer cells were observed as green fluorescent dots. The signal clearly decreased in wells containing A549 cells incubated with pulsed DCs, exo/pulsed DCs and exo/unpulsed DCs-primed alloPBMCs (at an E:T ratio of 50:1). In contrast, no change was observed in wells containing A549 cells incubated with unprimed-alloPBMCs. (**B**) Cytotoxicity in A549 cells induced by primed-alloPBMCs compared to that of unprimed-alloPBMCs. Pulsed DCs and exo/pulsed DCs primed-alloPBMCs were the most cytotoxic to A459 cells at all tested ratios. Interestingly, exosomes isolated from unpulsed DCs also induced alloPBMCs cytotoxicity against cancer cells. (**C**) Cytotoxicity in A549 cells induced by primed-alloT cells compared to that of unprimed-alloT cells. Both pulsed DCs and exo/pulsed DCs-primed alloT cells had greater effect on killing A549 cancer cells compared to unpulsed DCs at higher tested ratios. However, exosomes from unpulsed DCs showed no effect on the induction of alloT cell cytotoxicity. Data was presented as mean ± SD in triplicate cultures for (**B**) and twice cultures for (**C**). alloPBMCs: allogeneic peripheral blood mononuclear cells; alloT cells: allogeneic T cells; DCs: dendritic cells; pulsed DCs: A549 tumor cell lysate-pulsed DCs; unpulsed DCs: A549 tumor cell lysate-unpulsed DCs; Exo/unpulsed DCs: exosomes isolated from unpulsed DCs; Exo/pulsed DCs: exosomes isolated from pulsed DCs.

To analyze the cytotoxicity of alloPBMCs primed with DCs and their exosomes on A549 lung cancer cells, calcein-acetyoxymethyl (calcein-AM) cytotoxicity assay has been applied using TeraScan VPC2 (Minervatech, Japan). The results revealed a dramatic reduction in fluorescence signal after two hours of incubation of A549 cells with all DCs and pulsed DC-derived exosome-primed alloPBMCs and alloT cells in comparison with that incubation of A549 cells with unprimed ones (Figure 4A). This means that the cytotoxicity of primed-alloPBMCs and alloT cells were significantly higher than the unprimed (*p* < 0.05) (Figure 4B). Further analysis of the cytotoxicity test showed that the alloPBMCs and alloT cells primed with pulsed DCs had the highest cytotoxic activity against A459 cells at all tested E:T ratios. At a ratio of E:T of 50:1, pulsed DC-primed alloPBMCs and allT cells could kill 87% and 80%, respectively, of A549 cancer cells (Figure 4B). Additionally, exo/pulsed DCs showed 1.4- and 2-times greater effects on inducing cytotoxicity in alloPBMCs, and 2.4 times greater in allT cells at higher ratios of 25E:1T and 50E:1T, respectively, compared to exo/unpulsed DCs (Figure 4B). Exosome derived from unpulsed DCs shows no effect on the induction of alloT cells cytotoxicity at any tested ratio. Interestingly, these exosomes could induce alloPBMCs to kill A459 cells more efficient (1.4 times) than their parental cells at the highest ratio of E:T (Figure 4B). Taken together, these data indicated that all alloPBMCs primed with DCs or exosomes pulsed with A549 cancer cell lysate have greater effects on killing cancer cells.

## 3. Discussion

DCs play vital roles in anti-cancer immunity. However, the number of DCs, which is limited in cancer patients, is inefficient to mount immune responses against cancer cells in this manner [7]. AlloDC transplantation has been reported to induce T cell proliferation and antitumor immunity [5,6]. Thus, obtaining a reliable, readily available, and efficient source of DCs is necessary. AlloDCs and autoDC vaccination have been applied in several clinical trials, but to date there is no reports using cord blood monocyte-derived DCs and their exosomes in cancer treatment [7,8,9,10,11,12,13,14,15]. Cord blood (CB) stem cell transplantation can lower the rate and severity of graft-versus-host disease resulting from weak human leukocyte antigen (HLA) compatibility between donors and recipients [21]. Thousands of CB samples produced every day in hospitals can be used for many medical purposes, but it is difficult to obtain freshly isolated samples at facilities where blood transfusion is in demand. Thus, many studies have focused on the characteristics of cryopreserved CB specimens and then apply them for cancer management [20,21,26]. In this study, DCs were successfully generated from cryo CB in culture medium containing GM-CFS, IL-4, and TNF-α, which consistently showed a DC marker expression profile, such as CD80high, CD86high, CD40high, HLA-DRhigh, CD123low CD56low, and CD14low. Cryo CBMDCs expressed a typical DC morphology with thin and long dendrites, which have been described in previous reports [21,22,26,27,28,29,30]. Thus, our results indicate that cryo CB can be a potential source for DC generation, which then may be developed into immunotherapies. Furthermore, we successfully isolated exosomes from a CBMDC conditioned medium (morphology and immune phenotypic were proved in Figure 2A,B). Despite the limitations in this current study—e.g., the poor quality of Figure 2B due to the small amount of protein loaded in the immunoblotting experiment, which led to a week signal of exosomal marker—this data indicates that exosomes have been isolated successfully for the further investigation. Additionally, the immunoblotting data revealed that two exosomal markers investigated, including CD9 and CD63 were detected in parental cell lysates and each of the corresponding exosomes. However, CD63 were detected in exosomes, but not in their respective parental cells. The diverse enrichment of exosomal proteins as being dependent on the cell type from which the exosome is released has been previously reported [31,32].

Importantly, our results showed that allogeneic T cell proliferation was induced by both pulsed and unpulsed cryo CBMDCs. Previous studies provided evidences that tumor antigen-pulsed DCs can stimulate allogeneic T cells by increasing their antigen-presenting abilities compared to unpulsed DCs [33,34]. Moreover, Gruijl et al. presented the results of phase I clinical trial involving transplantation of an allogeneic DC line to treat acute myeloid leukemia [14]. They concluded that the treatment was safe, feasible, and could induce different levels of immune responses, even without HLA matching [15]. Thus, in this current study, we did not check the HLA phenotype of four DC samples and the responder allogeneic T cells. Besides, in this current study, CBMDCs were pulsed with whole A549 cell lysate in order that the entire repertoire of antigens associated with A549 cancer cells can be processed. This could prevent tumor immune escape through antigen-loss variants or mutations in critical T cell epitopes as some published studied reported [35]. It is noted that alloT cell proliferation increased three-fold in the group of the cells treated with pulsed DCs compared to the two-fold increase in the group of the cells treated with unpulsed DCs. Additionally, exosomes released from pulsed DCs have ability to enhance T cell proliferation, but with a lower capacity compared to their parental pulsed DCs. Unpulsed DC-derived exosomes have no effect on T cell proliferation despite the quite good effect of their parental unpulsed DCs. Previously, it has reported that several cellular mechanisms mediate information transfer between the T cell and the DCs, including transendocytosis, trogocytosis, formation of tunneling nanotubes, and polarized secretion of extracellular vesicles (EVs) [36]. Thus, in this current study, we did not perform exosome inhibition to limit the effects of exosomes secreted by DC in the incubation of DCs and T cells due to concerns that this exosome inhibition may not reflect a full DC function to proliferate T cells. Taken together, these data indicate that DCs have a better capacity to proliferate T cells than their secreted exosomes, in addition to DCs and DC-derived exosomes pulsed with A549 tumor lysate had a better antigen presentation capacity than that were not pulsed with A549 tumor lysate have.

An increase in the number of T cells including CD3^+^Vγ9 T cells and CD3^+^CD8^+^ T cell subsets is important to respond to pathogens. In this current study, tumor antigen-pulsed DCs had the highest effect on the induction of CD3^+^Vγ9 T cell and CD3^+^CD8^+^ T cell proliferation. Interestingly, pulsed DC-derived exosomes also had the capacity to proliferate these cell subsets. Previously, it showed that the astounding capacity of CD3^+^CD8^+^ T population to react to pathogens due to their massive expansion and differentiation into effector cells that specifically recognize infected or cancer cells [37]. Shortly after recognition of antigen on DCs, CD3^+^CD8^+^ T cells were activated and became cytotoxic T cells that continued to proliferate and antigen-specifically kill infected cells [37,38]. Based on these functions, CD3^+^CD8^+^ T cells became a potential target in immune cell therapy for cancer treatment. Recently, CD3^+^Vγ9 T cell population was shown to possess many antitumor characteristics in both in vitro [39,40,41] and in vivo studies [42,43,44], which enable them to have anti-tumor factors. However, the number of CD3+Vγ9 T cell population is rather small and accounted for less than 5% in peripheral blood [45,46]. Thus, this CD3+Vγ9 T cell population number needs to be increased in order to develop them into an effective therapy for antitumor immunity. The results from this current study showed a potential capacity of pulsed DCs and their exosomes to proliferate the T cells, including CD3+Vγ9 T cells in vitro.

In our study, both pulsed DCs and unpulsed DCs had ability in inducing T cells and T cell subset populations. Previous studies reported the immune activity of tumor antigen unpulsed DCs in inducing T cell growth and T cell activation [47,48]. DCs themselves had ability to induce T cell proliferation upon their interaction with the target cells and immature DCs can provoke antitumor activity [45,46]. The addition of antigen to DC therapy greater enhanced their function [47,48]. Consequently, pulsed DCs had a greater effect than unpulsed DCs have in the induction of T cell proliferation [33,34,47,48]. The results in our study indicated that the whole tumor cell lysate was success to activate CBMDCs. It worth noting that, recently, there is accumulating evidence that the release or exposure of damage-associated molecular patterns (DAMPs) also contributes to immunogenicity in vertebrates [49]. Some DAMPs interact to pattern recognition receptors (PRPs) (such as DNGR-1 in a good study by Sousa et al. [50]) may promote specific immunity against dead-cell-associated antigens by inducing DC activation. We need to do further experiments to reveal if whole A549 cell lysate can release DAMPs that contribute to the maturation of CBMDCs. In the case of exosomes secreted by pulsed DCs, these exosomes were proven to have either tolerogenic or immune-stimulatory effects when interacting with T cells [36,51,52,53]. In this current study, pulsed DC-derived exosomes could efficiently prime naïve T cells when directly interacting with target cells through incubation. Our data are consistent with published data about DC-derived extracellular vesicular, which can present antigen and carry costimulatory molecules, and can thus activate primed cognate T cells directly in the absence of activated bystander DC [4,5,6].

Previous studies reported that DCs induce not only T cells but also natural killer (NK) cells to kill cancer cells [4,27,33,34,38,51,52,53]. That led us to design the cytotoxicity assay using alloPBMCs rather than just using alloT cells as the only effector cells. Our findings indicated that DCs and their exosomes could induce the cytotoxicity of not only alloT cells but also alloPBMCs towards A459 cancer cells. AlloPBMCs and alloT cells primed with pulsed DCs expressed the highest anti-tumor activity with approximately 87% and 80%, respectively, of cancer death with the E:T cell ratio of 50:1. Additionally, alloPBMCs primed with pulsed DC-derived exosomes exerted higher anti-tumor cytotoxicity, with >70% of A549 cells killed, at a the E:T cell ratio of 50:1 compared to PBMCs primed with unpulsed DC-derived exosomes. Exosome isolated from unpulsed DCs had no effect on alloT cell cytotoxicity, but a similar cell proliferation allotment. Interestingly, unpulsed DC-derived exosomes could prime alloPBMCs to kill A549 cancer cells. That means these exosomes may have ability in the cytotoxicity induction of other immune cells, such as NK cells. Similar to this current study, many studies found that the capacity of pulsed DC-derived exosomes to induce CD8^+^ T cell proliferation in vitro and in vivo was stronger than that of unpulsed DC-derived exosomes, which then further induced tumor resistance-specific cytotoxicity against the T lymphocytes and their anti-tumor immunity [54,55]. In addition, pulsed DC-derived exosomes showed the capacity of priming naïve T cells and activating NK cells to attack tumor cells [38,53,54,55]. Therefore, exosomes released by DCs have been considered for the use as novel strategies for cancer treatment [22]. As mentioned, DCs can communicate with target cells. For example, in this current study, an incubation between DCs and PBMCs and T cell went downstream to induce cytotoxicity of cancer cells through exosome secretion [36]. Thus, exosome inhibition was not used in order to investigate the potential of DCs in priming alloPBMC and T cells.

Although autoDCs and their exosomes have been used in several clinical trials for cancer treatment, they have some limitations. Collecting autoDCs can cause harm to patient health [7,8,9,10,11,12,13,14,15,23,24,25]. The alternative source of DCs is alloDCs, but using alloDCs from donor’s blood also presents an ethical issue [7,8,9,10,11,12,13,14,15]. To address this problem, alloDC-derived exosomes have been investigated and showed a potential to replace alloDCs for cancer treatment [23,24,25]. Together with the current results of exosome effectively primed naïve T cells, it should consider DC-derived exosomes is an alternative therapy for cancer treatment. Thus, our study may open an innovative and efficient source of cryo CBMCs for the production of DCs and DC-derived exosomes which are promising for cancer treatment. However, there are still some points that need to be addressed, such as what about the antitumor immunity induction ability of CBMDCs compare to DCs derived from peripheral blood monocytes, and three primary DC subsets in blood: blood dendritic cell antigen (BDCA)-1, BDCA-2, and BDCA-3 [56,57]? Do the CBMDCs express any markers associated with conventional DCs such as XCR1 (X-C motif chemokine receptor 1), CD1c, CLEC10a (Ca2+-dependent lectin-type receptor family member 10A, CD301), and CD141? Since these markers are the indicators of conventional DC function and activation [56], it is necessary to figure out the presence of them on CBMDCs.

In conclusion, our findings demonstrated that alloDCs originated from cryo CBMCs and their secreted exosomes could induce immune responses against A549 lung cancer cells in vitro. Cryo CBMCs, which is banked, is readily and available for use to generate effectively DCs. These cryo CBMC-derived DCs and their exosome are potential to develop into a novel therapy such as vaccination and cancer treatment.

## 4. Materials and Methods

### 4.1. Materials

The protocols were reviewed and approved by the Ethics Committee of the Dinh Tien Hoang Institute of Medicine (document number IRB.009) on June 20, 2018. All methods were performed in accordance with the relevant guidelines and regulations. The mothers and the PBMC donors were informed about the purposes of this study. All subjects gave written informed consent in accordance with the Declaration of Helsinki.

This study was conducted with four umbilical cord blood (CB) units that met the requirements for cryopreservation at the private cord blood bank at Vinmec International Hospital in Vietnam from July to December 2018 [58]. Transmissible diseases were tested before blood collection. To be eligible, the blood had to be negative for human immunodeficiency virus, hepatitis B virus, hepatitis C virus, cytomegalovirus, and syphilis.

### 4.2. Thawing Cryopreserved CB Units and Mononuclear Cell Isolation

Four umbilical cord blood (CB) units from four different donors were removed from storage in liquid nitrogen, placed in a sterile plastic bag, and immediately immersed into a 37 °C water bath to thaw them rapidly (usually less than 2 min). The CB units were partially thawed until they had a slushy consistency and then diluted with an equal volume of culture medium RPMI 1640 (Gibco, Grand Island, NY, USA) supplemented with 20% (v/v) fetal bovine serum (FBS) (Gibco, Grand Island, NY, USA). Each suspension was then mixed and centrifuged at 400× *g* for 10 min at 4 °C. The supernatants were discarded and the sedimented cells were re-suspended in 20% (v/v) FBS in phosphate-buffered saline (PBS) with a pH of 7.4 (Gibco, Grand Island, NY, USA) for umbilical cord blood mononuclear cells (CBMCs) collection in the next step.

CBMCs or peripheral blood mononuclear cells (PBMCs) were purified by Ficoll-Paque (1.077 ± 0.001 g/mL, GE Healthcare, USA) via density-gradient centrifugation. Briefly, CB cells collected from Section 2.1 was centrifuged for 20 min at 840× *g* and 4 °C with low acceleration, followed by deceleration with no braking at room temperature. The buffy coat was collected then washed two times with PBS containing 20% (v/v) FBS.

### 4.3. Generation of DCs

CBMCs were seeded into T75 flasks containing KBM 551 lymphocyte serum-free medium (Corning Inc.) at a density of 2 × 10^6^ cells per mL. After 2 h of incubation at 37 °C in a humidified incubator with 5% CO_2_, the non-adherent cells were removed. The adherent cells were continuously cultured in KBM 551 medium containing recombinant human granulocyte-macrophage colony-stimulating factor (GM-CSF) (PeproTech Inc., Rocky Hill, NJ, USA) and interleukin 4 (IL-4) (PeproTech Inc., Rocky Hill, NJ, USA) at final concentrations of 1000 U/mL and 500 U/mL, respectively. Half of the culture medium was replaced with fresh medium containing cytokines every three days. On the 5th day, half of cell culture flasks were added A549 tumor cell lysate to a final protein concentration of 50 µg/mL and incubated to day 7. Both the cells added or not added with A549 cell lysate were supplemented with TNF-α (500 U/mL; PeproTech Inc., Rocky Hill, NJ, USA) on day six. All the cells were cultured for total seven days and harvested all the cells and conditioned media. The cells, which were not added A549 tumor cell lysate harvested at day seven, were named A549 cell lysate-unpulsed DCs (hereafter, ulpulsed DCs). The cells, which were added A549 tumor lysate harvested at day seven, were named A549 cell lysate-pulsed DCs (hereafter, pulsed DCs). The conditioned supernatants harvested on day seven were further used for exosome isolation.

For phenotypic analysis, the cell phenotypes were analyzed by flow cytometry. Monoclonal antibodies (mAbs) against CD3, CD8, Vγ9TCR, CD4, CD40, CD80, CD86, HLADR, CD123, CD14, and CD11c, as well as the corresponding isotype-matched controls were purchased from Beckman Coulter (Marseille, France). The mAbs were conjugated with Pacific Blue, fluorescein isothiocyanate, APC Vio, PC7, and Allophycocyanin- Alexa Flour 750 (APC-Alexa Flour 750). Cells were analyzed using a Navios flow cytometer (Beckman Coulter, Miami, FL, USA) and the data were acquired with Navios software, version 3.2 (Beckman Coulter, Miami, FL, USA).

### 4.4. A549 Lung Carcinoma Cell Culture and Preparation of Tumor Cell Lysates

A549 lung carcinoma cell lines were cultured in Dulbecco’s modified Eagle’s medium (Gibco Grand Island, NY, USA) containing 10% (v/v) FBS for later use as a source of tumor proteins. The A549 cells also served as target cells in the cytotoxicity assays.

A549 cells (4 × 10^7^) were subjected to at least ten freeze-thaw cycles to obtain a crude cell lysate. Freezing was performed by transferring the cells to liquid nitrogen and thawing was performed in a 37 °C-water bath. Large particles generated during freeze-thaw cycling was removed by centrifugation (2000× *g* for 10 min at 4 °C) and sterilized using a 0.22-µm filter. Protein concentrations were determined using Bradford protein assays and then aliquoted before storage at −80 °C for further use.

### 4.5. Exosome Isolation

Exosomes were isolated by differential ultracentrifugation as previously described [21]. Briefly, DC culture medium was collected and centrifuged at 300× *g* for 10 min at 4 °C and then the supernatant was collected. Each supernatant was centrifuged at 2000 g for 10 min at 4 °C before being transferred to a new tube and centrifuged for 30 min at 10,000× *g*. The supernatant was collected to a new tube and centrifuged for 70 min at 100,000× *g* (Optima XPN-100 Ultracentrifuge, Beckman Coulter, Brea, CA, USA) to obtain exosome pellets. The pellet was then re-suspended in PBS and centrifuged again at 100,000× *g* for 70 min at 4 °C to obtain cleaned exosomes. The cleaned exosomes were re-suspended in 50 µL PBS and either used immediately or stored at −80 °C for further use.

### 4.6. Analysis of Exosome Morphology via Transmission Electron Microscopy and Western Blot Analysis

Cleaned exosomes were fixed with 4% paraformaldehyde, after which a 5-µL drop of exosome suspension was loaded on a Formvar-carbon coated grid (TED PELLA, Inc., Redding, CA, USA) and allowed to dry naturally for 20 min. Grids were subsequently washed with PBS and incubated in 1% glutaraldehyde for 5 min before being washed 8 × 2 min with distilled water. Subsequently, samples were stained with uranyl-oxalate (pH 7) for 5 min at room temperature. Then, grids were transferred to methyl cellulose-uranyl acetate for 10 min on ice and allowed to dry at room temperature. Finally, exosomes were observed by a transmission electron microscopy (TEM) using a JEM1010 instrument at 80 KV (JEOL, Tokyo, Japan).

In Western blot analysis, for each sample, 5 µg exosomal proteins and cell lysate were loaded per lane and separated by 4–12% polyacrylamide SDS-PAGE gel (Invitrogen, Carlsbad Buck, UK) at 200 V for 35 min at 4 °C. Proteins were then transferred to PVDF membrane (GE Healthcare Life Science, Freiburg, Germany) at 200 mA for 2 h at 4 °C. The membrane was probed with antibodies (Santa Cruz Biotechnology, Dallas, TX, USA) against CD63 and CD9 (exosomal markers), CD86 (DC marker) (Beckman Counter, Marseille, France), and GAPDH followed by incubation with a horseradish peroxidase-conjugated anti-mouse antibody (Amersham^TM^, Freiburg, Germany). Proteins were detected with a chemiluminescence substrate (Amersham^TM^), and images were captured using an ImageQuant LAS 500 camera (GE Healthcare Life Sciences, Freiburg, Germany).

### 4.7. Allogeneic T Cell Purification and Proliferation Assay

CD3^+^ T cells from healthy donors’ PBMCs were separated via positive selection using the CD3 Microbeads Human Kit (MACS, Miltenyi Biotec Inc., Cologne, Germany). CD3 microbeads were added to cell suspensions and incubated for 15 min at 4 °C to label the CD3^+^ T cells. The cells were washed and then re-suspended in PBS (pH 7.4, Gibco, Marseille, France) containing 0.5% bovine serum albumin and passed through a column fastened to a magnetic separator. The cells were then allowed to flow into a collection tube and this cell population comprised of the unlabeled cell fraction of the total cells. The column was removed and flushed with a buffer to obtain an enriched CD3^+^ T cell population. Acquired T cell populations were characterized using flow cytometry. Cell counts and viability were determined. The cells were centrifuged, re-suspended in PBS, and aliquoted to 6 × 10^5^ cells/100 µL.

We evaluated the ability of cryo CBMDCs from four healthy donors and their exosomes to stimulate the proliferation of peripheral T cells. The T cells were stained with green fluorescence carboxyfluorescein succinimidyl ester (CFSE) (Thermo Scientific, Eugene, OR, USA). Then, 6 × 10^5^ CSFE-labeled T cells were seeded per well in a 12-well flat-bottomed plate. Then DCs or DC-derived exosomes were mixed with T cells with a ratio of 1 DC:4 T cell in a total volume of 1 mL KBM medium containing 50 U/mL IL-2 per well and cultured for four days at 37 °C and 5% CO_2_. The DC-derived exosome concentration that is equivalent to the number of secreting DCs were used to pulse the T cells (exosomes equivalent to 1 parental DCs:4 T cells). After incubation, the CFSE fluorescence intensity (FI) was measured using a Navios flow cytometer to evaluate T cell division.

### 4.8. Allogeneic T Cell Subset Proliferation Assay

The proliferation of allogeneic T cell subsets was also evaluated. The purified T cells were mixed with DCs or DC-derived exosomes were at a ratio of 1 DC:4 T cell in a total volume of 1 mL KBM medium containing 50 U/mL IL-2 per well and cultured for four days at 37 °C and 5% CO2. The DC-derived exosome concentration that is equivalent to the number of secreting DCs were used to pulse the T cells (exosomes equivalent to 1 parental DCs:4 T cells). After incubation, the floating cells were collected and stained with monoclonal antibodies against CD3, CD8, Vγ9TCR, and CD4 conjugated with Pacific Blue, FITC, APC Vio, and PC7 (Beckman Coulter, Marseille, France). Cells then were analyzed on BD FACScanto II system, and the data were acquired with BD FACSDiva^TM^ software (San Jose, CA, USA).

### 4.9. Cytotoxicity Assay

AlloPBMCs and allogeneic purified T cells (alloT cells) were primed with DCs with a ratio of 1 DCs:4 alloPBMCs/alloT cells and DC-derived exosomes equivalent to 1 DCs:4 alloPBMCs/alloT cells and cultured in KBM medium containing 50 U/mL IL-2. The primed alloPBMCs/alloT cells cultured for four day at 37 °C and 5% CO_2_ were used as effector cells (E). The A549 used as target cells (T) were labeled by incubating with Calcein-AM (Dojindo Molecular Technologies, Inc., Kumamoto, Japan) for 30 min at 37 °C and followed by a wash with PBS. The labeled A549 cells were then seeded into a 96-well flat-bottomed plate with a density of 3 × 10^3^ cells/well in Roswell Park Memorial Institute (RPMI) 1640 medium containing 10% FBS. Effector cells were then added to the target cells at three different E:T ratios of 12.5:1, 25:1, and 50:1. The plate was centrifuged for 2 min at 50× *g* in order to settle all the cells down to the bottom of the plate, then the fluorescence intensity (FI) was first measured using a Terascan VPC2 counter. For the maximum dye-release control, after taking the fluorescence measurement, the alkaline reagent DCN90 was added to the control wells. Then, the cells were incubated for 2 h. After incubation, the plate was centrifuged for 2 min at 50× *g*, the supernatant (80 µL) was discarded and 80 µL fresh RPMI was added into each well. The plate was centrifuged again for 2 min at 50× *g*, and a second measurement was taken on the Terascan VPC2 counter. The cytotoxicity (%) was calculated using the following formula, where the experimental value is the combined fluorescence of E + T, the spontaneous value is the FI of T + medium, and the maximum (positive control) value is the FI of T + DCN90:

(1) $$\%\;\mathrm{cytotoxicity}\;=\;\frac{\mathrm{experiment}\;-\;\mathrm{negative}\;\mathrm{control}\;}{\mathrm{positive}\;\mathrm{control}\;-\;\mathrm{negative}\;\mathrm{control}}\times 100$$

### 4.10. Statistical Analysis

Statistical analyses were performed with Stata 12.0 to determine *p*-Value using the paired/two-tailed Student’s *t*-test. A value of *p* < 0.05 was considered to statistically significant difference.

## Figures and Tables

**Table 1:** Incubation of DCs and their exosomes with T cells increased the proportions (%) of CD3^+^Vγ9 T cells, CD3^+^CD8^+^ T cells, and CD3^+^CD4^+^ T cells.

	CD3^+^Vγ9 T Cells	CD3^+^CD8^+^ T Cells	CD3^+^CD4^+^ T Cells
**Day 0**	5.2 ± 1.7	25.8 ± 4.2	35.2 ± 7.4
**T cells only**	5.7 ± 2.5	22.4 ± 7.4	36.7 ± 7.8
**Unpulsed DCs**	31.2 ± 3.1 *	55.3 ± 2.4 *	20.5 ± 11.2 *
**Exo/unpulsed DCs**	6.8 ± 1.2	30.4 ± 8.9	48.2 ± 8.6
**Pulsed DCs**	35.7 ± 7.4 *	52.7 ± 5.1 *	19.2 ± 5.8 *
**Exo/pulsed DCs**	32.1 ± 5.8 *	58.9 ± 4.6 *	15.7 ± 2.5 *

* *p* < 0.05.

## Abbreviations

DCs	Dendritic cells
CB	Cord blood
MNCs	Mononuclear cells
CBMCs	Cord blood mononuclear cells
AutoDCs	Autologous dendritic cells
AlloDCs	Allogeneic dendritic cells
EVs	Extracellular vesicles
CSFE	Carboxyfluorescein succinimidyl ester
Calcein-AM	Calcein acetoxymethyl
FI	fluorescence intensity
HLA	human leukocyte antigen
NK cells	Natural killer cells
GM-CSF	Granulocyte-macrophage colony-stimulating factor
IL	Interleukin
TNFα	Tumor necrosis factor α
TEM	Transmission electron microscopy
FBS	fetal bovine serum
DMEM	Dulbecco’s modified Eagle’s medium
RPMI	Roswell Park Memorial Institute medium
PBS	phosphate-buffered saline
